# Uterine fibroids in pregnancy: prevalence, clinical presentation, associated factors and outcomes at the Limbe and Buea Regional Hospitals, Cameroon: a cross-sectional study

**DOI:** 10.1186/s13104-018-4007-0

**Published:** 2018-12-13

**Authors:** Thomas Obinchemti Egbe, Therese Gaelle Badjang, Robert Tchounzou, Eta-Nkongho Egbe, Marcelin Ngowe Ngowe

**Affiliations:** 1 Department of Obstetrics and Gynaecology, Douala General Hospital, P.O. Box 4856, Douala, Cameroon; 2 Faculty of Health Sciences, University of Buea, P.O.Box 63, Buea, Cameroon; 3 Faculty of Medicine and Biomedical Sciences, University of Yaoundé 1, Yaoundé, Cameroon; 4 Department of Obstetrics and Gynaecology, Faculty of Health Sciences, University of Buea, Buea, Cameroon; 5 District Hospital Konye, South West Region, Konye, Cameroon

**Keywords:** Uterine fibroid, Pregnancy, Outcome, Cameroon

## Abstract

**Objectives:**

Uterine fibroids are common among the black race and associated with adverse outcomes in pregnancy. The aim of this study was to determine the prevalence, clinical presentation and maternal and foetal outcomes of birth among pregnant women with leiomyoma in two secondary care hospitals in Limbe and Buea, Cameroon.

**Results:**

The prevalence of fibroid in pregnancy was 16.7%. Respondents with leiomyoma were older than those without (p < 0.001) and of low parity (p = 0.02). Acute abdominal pain, (OR 3.8; 95% CI 1.4–9.9, p = 0.007), vaginal bleeding (OR 5.2; 95% CI 1.6–16.3, p = 0.004) were clinical presentation of leiomyoma in pregnancy. Cesarean birth (OR 4.5; 95% CI 1.4–13.6, p = 0.008), low Apgar score, (OR 6.0; 95% CI 1.9–19.1, p = 0.002), and postpartum hemorrhage (OR 4.7; 95% CI 1.7–13.2, p = 0.003) were adverse outcomes recorded.

**Electronic supplementary material:**

The online version of this article (10.1186/s13104-018-4007-0) contains supplementary material, which is available to authorized users.

## Introduction

Uterine fibroids are common amongst black women, have an earlier onset and are usually multiple and with higher complication rates than among white women [1]. The prevalence of uterine fibroids diagnosed by first trimester ultrasound was 18% in African American women [2] and 12.5% in Benin [3].

The few studies in Cameroon reported increased adverse obstetric outcomes among pregnant women with fibroids compared with those without [4].

Advances in ultrasonography have led to improved diagnostic criteria for uterine fibroids in pregnancy. In particular, these advances have impacted: (1) operational definitions that require a large diameter to define the presence of uterine fibroids (2) inconsistent documentation of uterine fibroids in clinical data used for research (3) difficulty detecting uterine fibroids as pregnancy progresses since uterine anatomy, foetus and placenta interfere with complete assessment of the myometrium.

Uterine fibroids are common among antenatal care attendants and parturients at the Limbe and Buea Regional Hospitals (LRH and BRH), Cameroon. However, we are not aware of any study regarding the association between uterine leiomyoma and pregnancy in the South West Region of Cameroon. We aimed at determining the prevalence, clinical presentation and maternal and foetal outcomes of birth among pregnant women with leiomyoma in two secondary care hospitals in Limbe and Buea, Cameroon.

## Main text

### Methods

#### Study design and setting

This was a cross-sectional study of pregnant women attending antenatal care at the Limbe and Buea Regional Hospitals in Cameroon during the period 1st April 2013 to 31st December 2013.

The LRH (birth rate of 1200 births per year) and BRH (birth rate 1050 births/year) are two secondary healthcare facilities that serve as referral centres in the South West Region of Cameroon and equipped to provide comprehensive emergency obstetric and neonatal care (EmONC) [5, 6].

#### Data collection and analysis

With a 95% confidence interval and power of 80% and a pre-study estimate of the prevalence of fibroids in pregnancy of 18% among African-American women [2], we estimated the minimum sample size of 226 participants using the Lorenz formula [7].

Structured questionnaires (approved by the Review Board of the University of Buea) that were pretested (tested on 15 pregnant women 1 week earlier for content validity) were administered to pregnant women aged ≥ 21 years who signed a written informed consent form, with a viable singleton intrauterine pregnancy and whose antenatal care was at the study health facilities by convenient and consecutive sampling method. The respondents should have a baseline first trimester ultrasound scan with data on uterine fibroids on those having fibroids and were willing to carry pregnancy to term, with no intention of moving from Limbe or Buea over the next 1 year and had to speak English, Pidgin or French. The study was designed to enrol women in the third trimester of pregnancy ≥ 28 weeks of gestation. During the face-to-face interview, the interviewer gathered information regarding diagnosis and treatment of uterine fibroids prior to current pregnancy and the data was later confirmed by studying the medical records of the respondents. Data regarding: personal and socio-demographic information, past and present obstetric and gynaecologic history were recorded. We also recorded maternal weight, height and blood pressure, fundal height, abdominal circumference including foetal presentation, position and heart rate.

Ultrasonography information of fibroids was based on Muram criteria [8]. The fibroid were identified, their diameter was measured in three perpendicular planes; measurements were made two times and fibroid diameter was averaged and mapped into a uterine diagram. It was later categorised by location (fundus, corpus, isthmus or cervix), position (anterior, posterior, right, left), and type (sub mucous-any fibroid in contact with or distorting the uterine cavity without identifiable myometrium between the fibroids and the endometrium, intramural-within the myometrium, neither distorting the contour nor the cavity, sub serous-distorting the external contour of the uterus, and pedunculated-attached to the uterus with an identifiable stalk). Participants with multiple fibroids had each fibroid documented separately and foetal morphology; placenta and adnexa images were saved initially in still print images and later on digital images on USB and sent for review by study investigators.

Participants were also taught the symptoms and signs of labour and asked to report to the maternity in case of labour.

During labour/delivery, the questionnaire was completely filled: gestational age at delivery, induction/augmentation of labour or not, mode of delivery (vaginal or caesarean), and foetal presentation at delivery were recorded. Maternal blood loss was also recorded.

Parameters of the baby studied were: Apgar score, birth weight, length, head, chest and abdominal circumferences.

At the end of the study, basic post-partum care education was given to all the respondents (advice on breast-feeding, contraception, breast and vulvar hygiene etc.).

The data collected was coded, double entered and analysed using EPI INFO™ 3.5.1 software. The categorical data (number of fibroids, gravidity, parity, educational level and employment status) were presented as frequencies and percentages whereas continuous variables (age, gestational age, Apgar score, foetal birth weight, body mass index (BMI)) were expressed as means and standard deviations. We compared percentages between two groups using the student t-test and Chi squared test where appropriate and Odds ratios and their 95% confidence intervals were generated and significance level was set at p < 0.05.

### Results

During the study period, we approached 288 pregnant women; 18 (9.27%) did not consent to study and 44 (22.68%) were excluded because of lack of first trimester ultrasound scan. We were then left with 226 (68.04%) respondents who completed the study among which; 38 (16.7%) had fibroids and 188 (83.3%) were without fibroids (Additional file 1: Figure S1).

**Table 1:** General characteristics of participants

Variable	Over all (N = 226)	Percentage %	Fibroids (N = 38)	Percentage %	No fibroids (N = 188)	Percentage %	p-value
Age (years), mean (SD)	28.1	4.3	31.4	3.4	27.4	4.2	< 0.001
Age (years), n (%)							
20–25	45	19.7	0	0.0	44	23.6	0.001
25–30	108	47.7	14	36.4	94	50.0	
30–35	51	22.7	17	45.5	34	18.2	
35–40	22	9.8	4 (7)	18.2	16	8.2	
Education, n (%)							
Primary	41	18.2	5	13.6	36	19.2	0.90
Secondary	125	55.3	23	59.1	102	54.5	
Tertiary	60	26.5	10	27.3	50	26.4	
Age at menarche, mean (SD)	14.6	1.7	14.0	1.8	14.7	1.6	0.11
Oral contraceptive use, n (%)	45	19.7	8.63	22.7	36	19.1	0.77
Gravidity, mean (SD)	2.7	1.6	2.3	1.7	2.8	1.5	0.17
Gravidity, n (%)							
1	57	25.0	19	50.0	38	20.0	0.02
2	57	25.0	3	9.1	53	28.2	
3–4	87	38.6	14	36.4	73	39.1	
≥ 5	25	11.4	2	4.5	24	12.7	
Body mass index (kg/m^2^), n (%)							
<25	48	21.2	7	18.2	41	21.8	0.50
25–30	51	22.7	12	31.8	39	20.9	
30–34	106	47.0	14	36.4	92	49.1	
> 35	21	9.1	5	13.6	16	8.2	

%: Percentage

*SD* standard deviation

Respondents with uterine fibroid in pregnancy were older than those without (p < 0.001) and majority were in the age group 25–30 and 30–35 years (p = 0.001). They were mainly primiparous (p = 0.02)  (Table 1).

**Table 2:** Clinical features of uterine fibroids among respondents with fibroids

Variables	No. of patient (N = 38)	Frequency (%)
Size of largest fibroid (cm), mean (SD)	2.82 ± 1.64	1.8
Feeling of a mass	16	40.9
Type of fibroid		
Intramural	5	13.6
Submucous	9	22.7
Subserous	24	63.7
Location of fibroid		
Cervix	2	4.5
Fundus	29	77.3
Tubes	2	4.5
Pedunculated	5	13.6
Number of fibroids		
1	9	22.7
2–3	19	50.0
> 3	10	27.3

*SD* standard deviation

The mean size of fibroids was 2.82 (SD 1.64) cm and 41% respondents had a sensation of pelvic heaviness. Majority, 63.7% fibroids were sub-serous, 22.7% were sub-mucous and 13.6% intramural. About 22.7% respondents had ≤ 1 child while 50% had 2–3 children (Additional files 2, 3: Figures S2, S3 (Table 2).

**Table 3:** Uterine fibroids in pregnancy and outcomes

Outcomes	Fibroids				OR	95% CI	p-value
	Yes (N = 38)	Percentage %	No (N = 188)	Percentage %			
Acute abdominal pain, n (%)	17	45.5	34	18.2	3.8	1.4–9.9	0.007
Vaginal bleeding, n (%)	12	31.8	15	8.2	5.2	1.6–16.3	0.004
Miscarriage, n (%)	14	36.4	62	32.7	1.2	1.6–16.3	0.74
Delivery before 37 weeks, n (%)	5	13.6	17	9.1	1.6	03–5.7	0.51
Cesarean delivery (N = 128), n (%)	12	31.8	18	9.4	4.5	1.4–13.6	0.008
Baby weight < 2500 g, n (%)	5	13.6	9	4.5	3.3	0.6–14.7	0.12
5 min Apgar score ≤ 7, n (%)	12	31.8	14	7.3	6.0	1.9–19.1	0.002
Blood loss at delivery > 500 mL, n (%)	16	40.9	24	12.7	4.7	1.7–13.2	0.003

%: Percentage

*OR* odds ratios, *CI* confidence interval

Acute abdominal pain, (OR 3.8; 95% CI 1.4–9.9, p = 0.007), vaginal bleeding (OR 5.2; 95% CI 1.6–16.3, p = 0.004), Cesarean birth (OR 4.5; 95% CI 1.4–13.6, p = 0.008), low 5 min Apgar score, (OR 6.0; 95% CI 1.9–19.1, p = 0.002), and primary postpartum hemorrhage (OR 4.7; 95% CI 1.7–13.2, p = 0.003) were adverse outcomes associated with uterine fibroids in pregnancy (Table 3).

### Discussion

We determined the prevalence, clinical presentation and maternal and foetal outcomes of birth among pregnant women with leiomyoma in two secondary care hospitals in Limbe and Buea, Cameroon.

The prevalence of uterine fibroids in pregnancy was 16.7%. Increasing age (p = 0.0001) and low parity (p = 0.02) were associated with uterine fibroids in pregnancy. Feeling of pelvic mass (p = 0.05), acute abdominal pain (p = 0.005), vaginal bleeding (p = 0.002) were clinical features associated with uterine fibroids in late pregnancy.

Adverse maternal and foetal outcomes recorded were low 5-min Apgar scores, increased caesarean birth rates and primary post-partum haemorrhage.

#### Prevalence of uterine fibroids in pregnancy

The association of uterine fibroids and pregnancy has been reported in some African studies; Benin (12.5%) and Nigeria (12.3%), respectively [3, 9] and 0.87% was reported in Pakistan [10]. However, the prevalence reported in those studies was lower than the 16.7% found in our study. Our results are similar to those reported from two studies among African American women; 14.7% and 18% [2, 11]. These higher estimates are more compatible with those required to reach the prevalence documented in imaging studies of older non-pregnant women. It is worth noting that there is a lower incidence of leiomyoma’s detected by ultrasonography during pregnancy [12] and this varies according to the trimester of assessment and size threshold [13, 14]. Therefore, obstetricians should screen for leiomyoma early in pregnancy among Cameroonian (black race) because they are at risk of having fibroids than the white race. This is linked to the high levels of oestrogen receptors ER-α PP genotype found in black women as compared to white women [15].

#### General characteristics of respondents

The association of increasing frequency of uterine fibroids in pregnancy with advancing maternal age has been reported in other African studies [4, 9]. The reason for this association may be because women in Cameroon nowadays delay childbearing for educational and career purposes. Furthermore, all the respondents were of the black race and therefore, form a high-risk group for uterine fibroids.

The respondents in this study were mainly primiparous. The average household size in Cameroon has reduced from 6.0 persons per household in the 1960’s to about 5.2 per household in recent times and there are 3.2 children per household on the average [16, 17]. However, this low parity could not be attributed to the presence of uterine fibroids alone but to other co-morbidities like sexually transmitted infections, which are frequent in Cameroon [2, 4, 9]. The protective effect of multiparity on uterine leiomyoma has been reported previously. This was confirmed in our study and it has been postulated that this protective effect occurs during uterine involution where there is remodeling of the uterine smooth muscle [18].

#### Clinical features of fibroids in pregnancy and outcomes

The presence of pressure symptoms, abdominal pain and vaginal bleeding during pregnancy in our study is consistent with other studies that reported symptoms, like sensation of abdominal heaviness, and pressure symptoms (increased urine frequency), lower abdominal pain and vaginal bleeding during pregnancy [19–21]. Another study carried out in five countries in Europe reported that uterine fibroids were associated with impairment of health related quality of life [20]. Muran et al. have classified uterine fibroids according to types [8]. However, we found mainly sub serous (63.5%) and fundal (77.3%) leiomyoma’s in this study, which may create pressure symptoms; but pain is usually associated with ischemia due to growth of some fibroids during pregnancy and poor blood supply to the growing fibroid [20]. Vaginal bleeding has been reportedly associated with sub mucous fibroids or insertion of the placenta near a fibroid and may have an adverse impact on fertility [4, 9, 11, 18, 19]. Earlier studies have reported abruption placentae (57%), significant pain requiring narcotic analgesia (15.1%), preterm labor (21.5%) and increased rates of cesarean section among pregnant women with uterine fibroids [4, 9, 22–25]. This is consistent with our study that found low 5 min Apgar scores, increased rates of cesarean births and increased rates of primary post-partum hemorrhage. Therefore, obstetricians should be equipped for emergency cesarean birth, anticipate active management of third stage of labour on parturients with leiomyoma and be ready for resuscitation of the newborn.

### Conclusion

One in every six pregnant women had uterine leiomyoma that presented as a sensation of pelvic heaviness, lower abdominal pain and vaginal bleeding. The adverse maternal and fetal outcomes noted were low 5-min Apgar scores, increased rates of cesarean births and primary post-partum hemorrhage.

## Limitations

This paper provides supportive data on the local prevalence of fibroids in pregnancy but we could not draw any conclusions about the profile of fibroids or maternal or foetal outcomes because the numbers are too small. We did not assess the clinical experience of the sonographers for homogeneity before onset of study; since scan results were from different sonographers. We did not modify the criteria for diagnosis of uterine fibroid in pregnancy as in other studies; otherwise the prevalence may have been higher.

## Additional files

**Additional file 1: Figure S1.** Flow diagram.

**Additional file 2: Figure S2.** Exteriorized uterus with multiple uterine leiomyoma’s after extraction of fetus at Cesarean birth.

**Additional file 3: Figure S3.** Posterior view of exteriorized uterus with multiple uterine fibroids.

## Abbreviations

BMI: body mass index
BRH: Buea Regional Hospital
EmONC: Emergency Obstetric and Neonatal Care
LRH: Limbe Regional Hospital
OR: odd ratio
SD: standard deviation
USB: Universal Serial Bus
