# Supplementary material for: Uterine fibroids in pregnancy: prevalence, clinical presentation, associated factors and outcomes at the Limbe and Buea Regional Hospitals, Cameroon: a cross-sectional study
Source: BMC Res Notes. 2018 Dec 13;11:889. doi: 10.1186/s13104-018-4007-0 (PMC6293543; doi:10.1186/s13104-018-4007-0)
Supplement: Supplementary file 1 — Additional file 1: Figure S1. Flow diagram. [file 13104_2018_4007_MOESM1_ESM.docx]

**Number of Participants who completed study**

**= 226**

**Did not give consent to study**

**=18**

**Participants without fibroids**

**= 188**

**Participants with uterine fibroids**

**=38**

**Number of Participants Approached**

**= 288**

**Excluded because of lack of first trimester ultrasound and not willing to do it**

**= 44**

**Figure S1: Flow Diagram**
