# Supplementary figures and images for: Uterine fibroids in pregnancy: prevalence, clinical presentation, associated factors and outcomes at the Limbe and Buea Regional Hospitals, Cameroon: a cross-sectional study
Source: BMC Res Notes. 2018 Dec 13;11:889. doi: 10.1186/s13104-018-4007-0 (PMC6293543; doi:10.1186/s13104-018-4007-0)

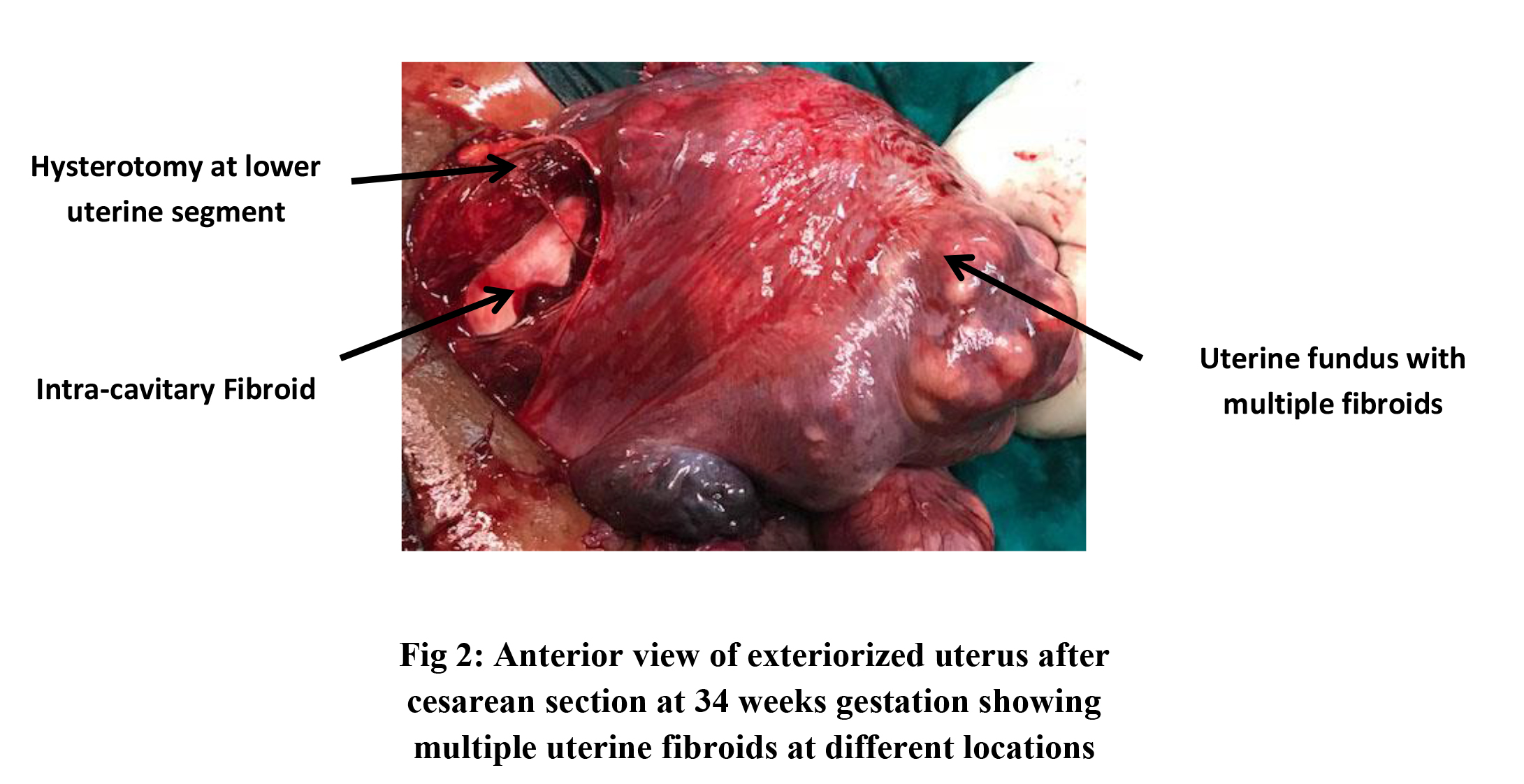

Supplement: Supplementary file 2 — Additional file 2: Figure S2. Exteriorized uterus with multiple uterine leiomyoma’s after extraction of fetus at Cesarean birth. [file 13104_2018_4007_MOESM2_ESM.jpg]

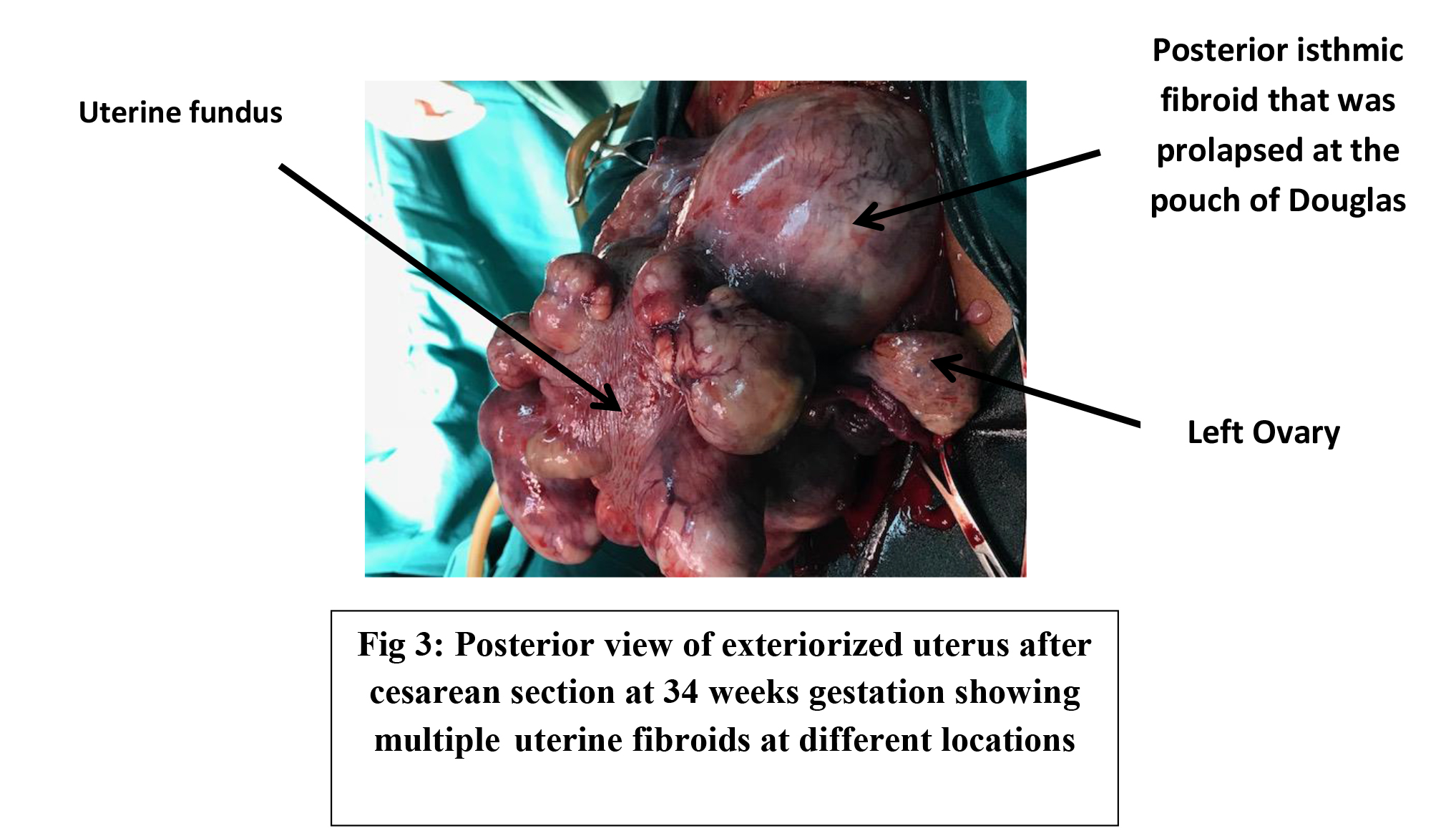

Supplement: Supplementary file 3 — Additional file 3: Figure S3. Posterior view of exteriorized uterus with multiple uterine fibroids. [file 13104_2018_4007_MOESM3_ESM.jpg]
